# Systematically Dissecting the Function of RNA-Binding Proteins During Glioma Progression

**DOI:** 10.3389/fgene.2019.01394

**Published:** 2020-01-28

**Authors:** Jianjun Wang, Jianfeng Qi, Xianzeng Hou

**Affiliations:** ^1^ Department of Neurosurgery, The First Hospital Affiliated with Shandong First Medical University, Shandong Provincial Qianfoshan Hospital, Jinan, China; ^2^ College of Medicine, Shandong First Medical University, Taian, China

**Keywords:** glioma progression, RNA-binding protein, mutations, regulatory network, prognosis

## Abstract

RNA-binding proteins (RBPs) play important roles in regulating gene expression and dysregulation of RBPs have been observed in various types of cancer. However, the role of RBPs during glioma progression, and particular in Chinese patients, is only starting to be unveiled. Here, we systematically analyzed the somatic mutation, gene expression patterns of 2949 RBPs during glioma progression. Our comprehensive study reveals several of highly mutated genes (such as ATRX, TTN and SETD2) and differentially expressed genes (such as KIF4A, TTK and CEP55). Integration of the expression of RBPs and genes, we constructed a regulatory network in glioma and revealed the functional links between RBPs and cancer-related genes. Moreover, we identified the prognosis spectrum of RBPs during glioma progression. The expression of a number of RBPs, such as SNRPN and IGF2BP3, are significantly associated with overall survival of patients in all grades. Taken together, our analyses provided a valuable RBP resource during glioma progression, and revealed several candidates that potentially contribute to development of therapeutic targets for glioma.

## Introduction

RNA-binding proteins (RBPs) play crucial roles in post-transcriptional events and perturbations in RBP activity have been associated with various types of cancer (Pereira et al., 2017; Hentze et al., 2018). Understanding the function of RBPs in cancer will help identifying potential prognostic and response biomarkers for design of therapeutic targets (Bonnal et al., 2012; Kudinov et al., 2017). Glioma is a common and aggressive type of brain tumor, which was with poor outcome and no effective treatment by far (Ostrom et al., 2014; Reifenberger et al., 2017). Systematical dissection of RBP functions during glioma progression will provide new insights into the underlying mechanisms of glioma.

Comprehensive identification and annotation of human RBPs are the primary step for investigating their functions. With the development of high throughput sequencing, numbers of RBPs have been identified (Gerstberger et al., 2014). Several databases have curated a number of RBPs. For example, RBPDB is a database for collection of experimentally validated RBPs (Cook et al., 2011). ATtRACT also manually curated approximate 370 RBPs (Giudice et al., 2016). Recently, EuRBPDB has been constructed, which is a widely-used resource for RBPs (Liao et al., 2019). Moreover, number of studies have found a lot of alterations in RBPs during cancer development and progression. Wang et al., revealed the importance of RBPs in carcinogenesis by large-scale transcriptional profiling studies (Wang et al., 2015). The mutational spectrum of RBPs had been analyzed and identified a number of RBPs exhibited significantly mutation in cancer (Neelamraju et al., 2018). Li et al., have also decoded the genome-wide RBP mutational and transcriptomic landscape and yielded valuable insights into the function of RBPs (Li et al., 2019a). All these results suggest that there are prevalent alterations of RBPs in cancer development and progression.

RBPs have also been demonstrated to play important roles in neurodegeneration and glioma progression (Pereira et al., 2017). Numbers of RBPs have also been identified in glioma. Correa et al. revealed the splicing regulator SNRPB as an oncogenic candidate in glioblastoma (GBM) through functional genomics analyses (Correa et al., 2016). RNA-binding protein PCBP2 has been identified to modulate glioma growth by regulating FHL3 (Han et al., 2013). Moreover, Musashi1 was found to be a central regulator of adhesion pathways in GBM (Uren et al., 2015). The RBP IMP2 can preserve GBM stem cells by preventing let-7 target gene silencing (Pereira et al., 2017). Although a number of regulatory networks have been analyzed during glioma progression, such as microRNA-gene regulatory network (Li et al., 2013), transcriptional regulatory network (Li et al., 2015), competitive endogenous RNAs (ceRNAs) network (Xu et al., 2015), we are still lack of knowledge about RBP regulatory network during glioma progression.

To address these questions, we systematically analyzed the genetic and transcriptomic alterations of RBPs during glioma progression. We identified a number of RBPs with somatic mutations, differentially expressed during glioma progression. In addition, several RBPs associated with patient overall survival were also identified. These RBPs regulated a number of cancer-related genes and played important roles during glioma progression in Chinese patients. All these results provide novel insights into the function of RBPs in glioma.

## Materials and Methods

### Collection of Human RNA-Binding Proteins

All the human RBPs were downloaded from the EuRBPDB database, which is a comprehensive resource for annotation of eukaryotic RBPs. In total, there were 2,949 RBPs and these RBPs were further classified into canonical and non-canonical RBPs.

### Genetic Alteration Profiles of Glioma

The genetic alterations for Chinese glioma patients were downloaded from Chinese Glioma Genome Atlas (CGGA). There were 286 patients sequenced by whole-exome sequencing (Hu et al., 2018). We directly downloaded the gene-level mutation datasets. In this table, each row represents one gene and the columns represent the glioma patient. The one values indicated that this gene was mutated in corresponding patient while zeros indicated not mutated. In addition, we also downloaded the clinical information of these patients from CGGA (**Table S1**). The grade, gender, age, overall survival time, censor status and isocitrate dehydrogenase (IDH) mutation status were included. In addition, we downloaded the somatic mutations of low-grade glioma (LGG) and GBM patients from The Cancer Genome Atlas (TCGA) project. The overall survival and disease-free survival time of these patients were also downloaded.

### Genome-Wide Gene Expression Profile of Glioma

Genome-wide gene expression of glioma patients were also downloaded from CGGA (September 9, 2019). mRNA-Seq data were used in our analyses, which included 693 patients in total. The reads were aligned by STAR (Dobin et al., 2013) and the expression were evaluated as RSEM (Li and Dewey, 2011). There were 185 patients with both somatic mutations and RNA-Seq data (**Figure S1**). We also downloaded the clinical information for these 693 patients (**Table S2**).

### Identification of Top Mutated Genes in Glioma

To identify the genes with high mutation frequency, we separately ranked each gene in grade II, III, and IV glioma. The gene mutation frequency was defined as:

$$F\left(g\right)=\frac{n_{g}}{N}$$

where *n_g_* was the number of patients with gene *g* mutated and *N* was the total number of patients in specific grade.

### Identified the Genes With Perturbed Expression in Glioma

We used Wilcoxon’s rank sum test to evaluate the difference of gene expression between two adjacent grades. For example, the fold changes for comparison between grade II and III were defined as the (average expression of genes in grade III)/(average expression of genes in grade II). The *p*-values of Wilcoxon’s rank sum test were adjusted by Benjamini–Hochberg (BH) method. Genes with fold changes > 2 and adjusted *p*-values < 0.05 were defined as up-regulated genes and those with fold changes < 0.5 and adjusted *p*-values < 0.05 were defined as down-regulated genes. The comparisons were performed between grade II vs. III, and III vs. IV.

### Construction of RNA-Binding Protein–Gene Regulatory Network in Glioma

RNA-binding proteins are key regulators of gene expression, yet only a small fraction have been functionally characterized (Pereira et al., 2017). It is still difficult to identify the target genes for the majority of RBPs. Increasing studies have demonstrated that the regulators are likely to co-express with their target genes. Thus, we identified the co-expressed genes of RBPs and constructed the RBP–gene regulatory network in glioma. Here, only the cancer genes were considered and the cancer-related genes were downloaded from COSMIC Cancer Gene Census (Oct 25, 2019) (Sondka et al., 2018). There are 723 genes in total. For each RBP–gene pair, we calculated the Pearson correlation coefficient (PCC) as follows:

$$R_{ij}=\frac{1}{n-1}\sum_{i=1}^{n}\left(\frac{X_{i}-\bar{X}}{\sigma_{X}}\right)\left(\frac{Y_{i}-\bar{Y}}{\sigma_{Y}}\right)$$

where $\bar{X}$nd $\bar{Y}$where the average expression of RBP *X* and gene *Y*, *σ*_*X*_ and *σ*_*Y*_where the standard error of expression of RBP and gene. There were *n* patients in the analysis. All the RBP–gene pairs with PCCs > 0.70 and *p*-values < 0.05 were identified to construct the regulatory network. The network was visualized by Cytoscape (version 3.7.1) (Shannon et al., 2003).

### Identifying Clinical-Associated RNA-Binding Proteins in Glioma

To identify the RBPs whose expression was potentially correlated with glioma patient survival, we first divided patients in each grade into two groups based on the median expression of each RBP. The survival difference between two groups were evaluated by log-rank test. The hazard ratio (HR) was also calculated. This procedure was performed by the R package (version 3.6.1) (https://cran.r-project.org/web/packages/survival/index.html). RBPs with HR > 1 and *p* < 0.05 were defined as risky factors and those with HR < 1 and *p* < 0.05 were defined as protective factors.

## Results

### High-Grade Glioma Patients Exhibit Poor Prognosis and Less Isocitrate Dehydrogenase Mutation

**Figure 1:**
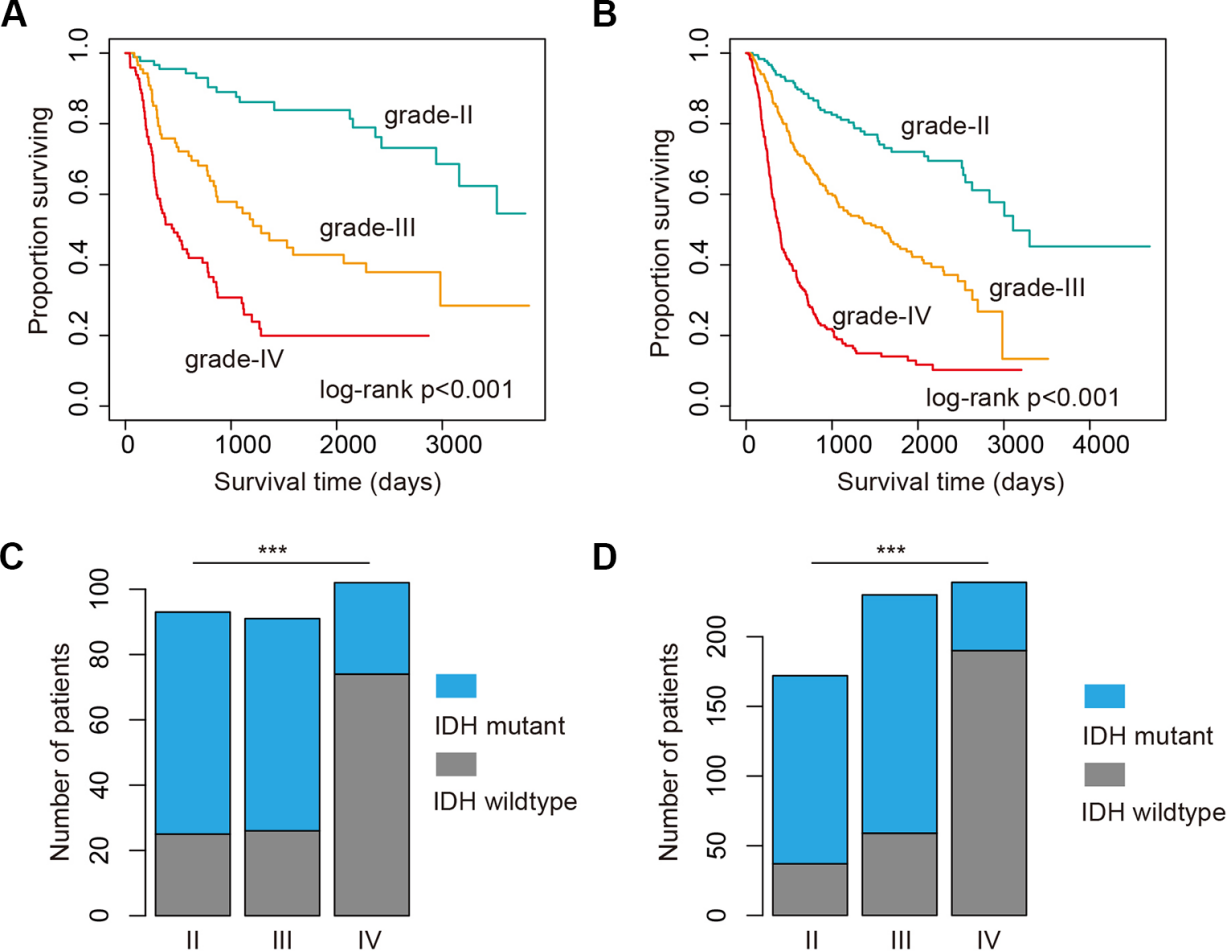
High grade glioma patients with poor survival and less isocitrate dehydrogenase (IDH) mutation. **(A)** Kaplan–Meier plot indicating survival of glioma patients with mutation data in different grades. **(B)** Kaplan–Meier plot indicating survival of glioma patients with expression data in different grades. **(C)** The proportion of patients with IDH mutation or wild type in different grades. Those patients were with mutation data. **(D)** The proportion of patients with IDH mutation or wild type in different grades. Those patients were with expression data. ***P < 0.001.

High-grade glioma remains incurable despite number of genetic alterations have been revealed (Chen et al., 2016). Here, we analyzed the 286 glioma patients sequenced by whole-exome sequencing. We found that glioma patients in high-grade exhibited poor prognosis (**Figure 1A**, log-rank *p* < 0.001). Moreover, we explored the 693 patients with mRNA-Seq data. We also found that the patients in high grade were with significantly poorer survival (**Figure 1B**, log-rank *p* < 0.001). Particularly, the patients in grade IV (glioblastoma, also known GBM) were with the poorest survival (**Figures 1A, B**). These results were consistent with the current knowledge, that GBM is the most aggressive cancer.

**Figure 2:**
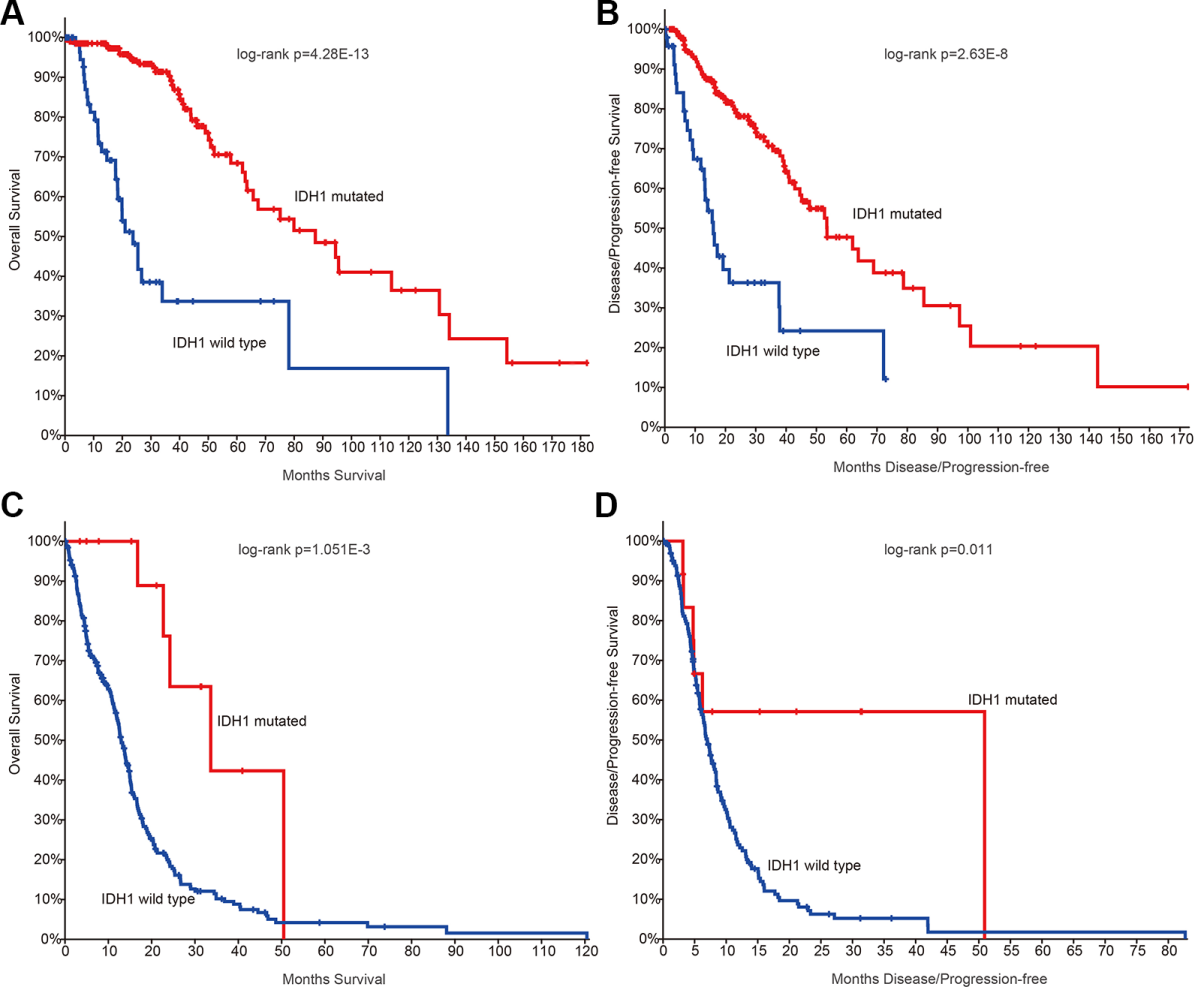
The survival plots for glioma patients in The Cancer Genome Atlas (TCGA) project. **(A)** Kaplan–Meier plot indicating overall survival of low-grade glioma patients with isocitrate dehydrogenase 1 (IDH1) mutation or not. **(B)** Kaplan–Meier plot indicating disease-free survival of low-grade glioma patients with IDH1 mutation or not. **(C)** Kaplan–Meier plot indicating overall survival of glioblastoma (GBM) patients with IDH1 mutation or not. **(D)** Kaplan–Meier plot indicating disease-free survival of GBM patients with IDH1 mutation or not.

Next, we investigated whether there are somehow difference in the clinical information for patients in different grades. We first compared the ages of patients with mutation data. The average ages for patients in grade II and III were 38.21 and 39.53 years. The average ages for patients in grade IV were 47.56, which were significantly older than II and III (*p*-values < 0.01, Wilcoxon’s rank sum tests). Moreover, we got the similar results in the patients with mRNA data. However, there were no significant difference between grade II and III. IDH1 is the most commonly mutated gene in glioma (Philip et al., 2018). We thus investigated the mutation frequency of IDH1 in glioma patients. We found that GBM patients were with less IDH mutation, either in the exome sequencing cohort or the mRNA-Seq cohort (**Figures 1C, D**, *p*-values < 0.001, Fisher’s exact test). Moreover, we analyzed the data from TCGA project and found that patients with IDH1 mutation exhibit better overall survival than the wide type ones in LGG and GBM (**Figures 2A, C**). When considering the disease-free survival time, we found that patients with IDH1 mutation also show better survival in LGG and GBM (**Figures 2B, D**). All these results suggest that high-grade glioma patients were older, were not likely with IDH1 mutation and exhibited poor survival.

### Prevalent Somatic Mutations of RNA-Binding Protein During Glioma Progression

**Figure 3:**
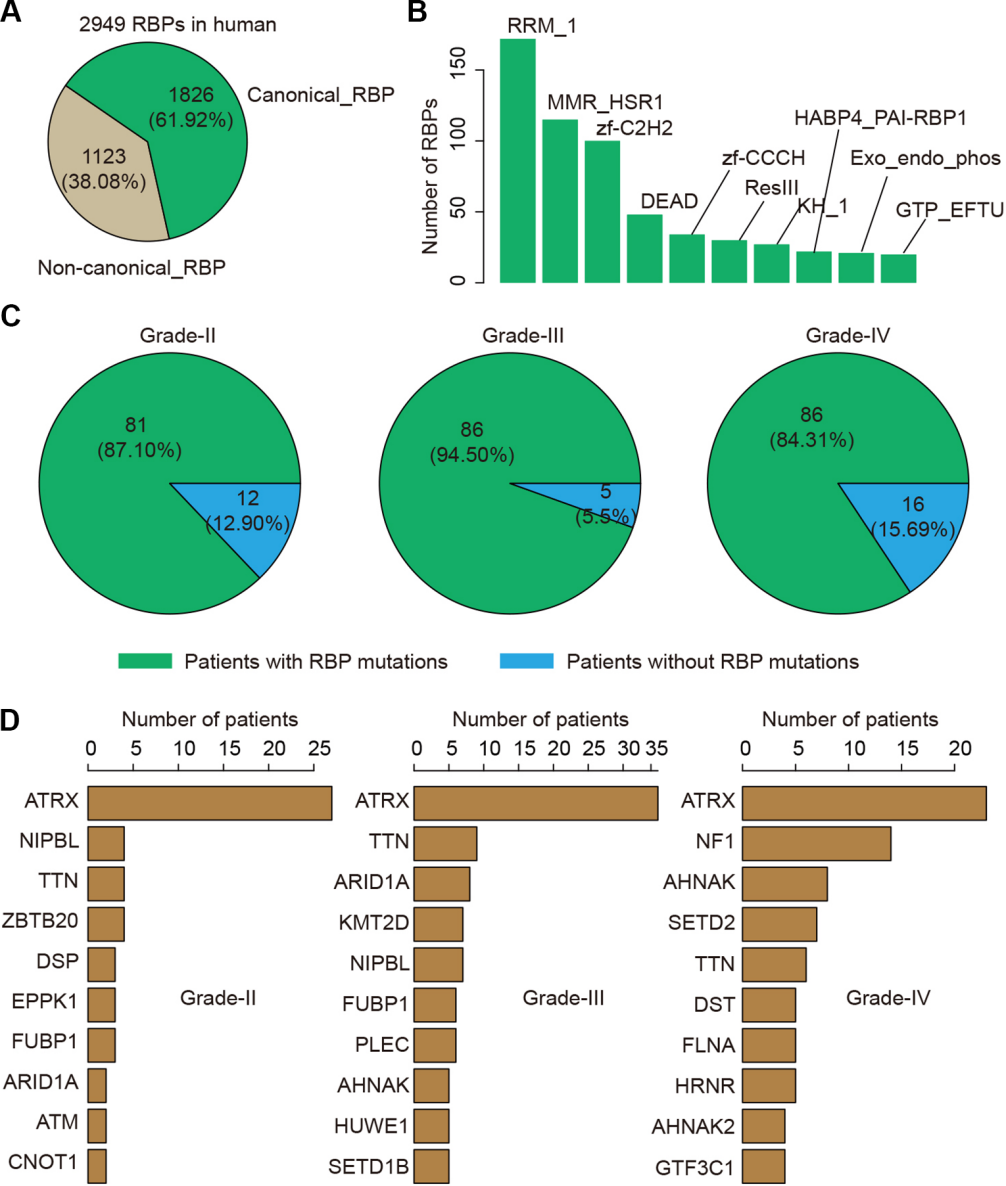
The mutation spectrum of RNA-binding protein (RBP) mutations in glioma. **(A)** The pie chart shows the proportion of canonical and non-canonical RBPs. **(B)** The bar charts shows the number of RBPs in top ranked RBP families. **(C)** The pie charts show the proportion of patients in different grade with RBP mutations. Left for grade II, middle for grade III and right for grade IV. **(D)** Top ranked 10 genes by mutation frequency in different grades. Left for grade II, middle for grade III and right for grade IV.

RBPs have been found to play critical roles in glioma. We thus next investigated the genetic alterations of 2,949 RBPs in glioma (**Figure 3A**). There were 1,826 (61.92%) canonical RBPs with specific RNA binding domains, and 1,123 (38.08%) non-canonical RBPs. Next, we calculated the number of RBPs with different binding domains. We found that there were more than 150 RBPs with RRM_1 domains (**Figure 3B**). We explored whether each patient was with RBP mutation and found that approximate 87.10% patients in grade II, 94.50% patients in grade III and 84.31% patients in grade IV were with RBP mutations (**Figure 3C**). These results suggest that there were prevalent somatic mutations in RBPs during glioma progression.

**Figure 4:**
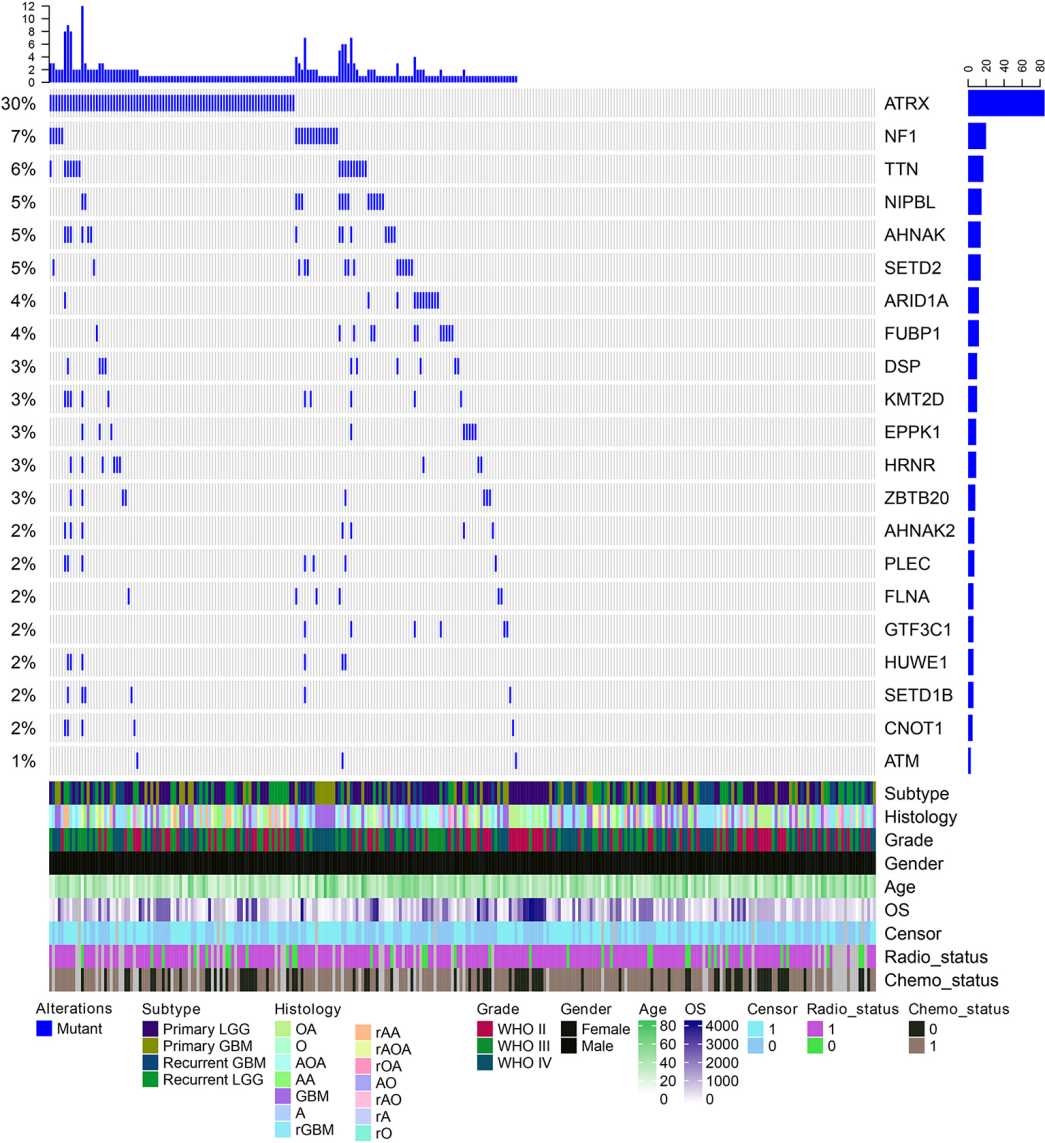
The mutation spectrum of top ranked RNA-binding proteins (RBPs) in glioma. Each column is one glioma patient. The blue lines indicate whether the gene is mutated or not. The clinical information of these patients are shown on the bottom panels.

We next explored which RBPs were with higher mutation frequency in different grades glioma patients. Top ranked 10 mutated genes in each grade were shown in **Figure 3D**. We found that ATRX was ranked top 1 in all grades. The ATRX status has been found to be one of the critical markers that define the molecular classification of gliomas (Nandakumar et al., 2017). ATRX loss can promote tumor growth and impair DNA repair in glioma (Koschmann et al., 2016). In total, we found that ATRX1 was mutated in approximate 30% of all glioma patients (**Figure 4**). Another frequently mutated gene was TTN in all grades (**Figure 3D**), which was also identified previously in glioma (Panossian et al., 2018). TTN was mutated in 6% of all glioma patients (**Figure 4**). Moreover, we found that NF1 were with higher mutation frequency in GBM, which has been used to define the mesenchymal subtype of GBM (Verhaak et al., 2010). We also identified several candidate genes, such as ARID1A, SETD2, FLNA and KMT2D. In addition, we queried the PubMed and found that numbers of these genes were co-occurred with “glioma” or “glioblastoma” in literature (**Figure S2** and **S3**). These results provided candidate RBPs for further functional investigation in glioma.

### Expression Perturbations of RNA-Binding Proteins During Glioma Progression

**Figure 5:**
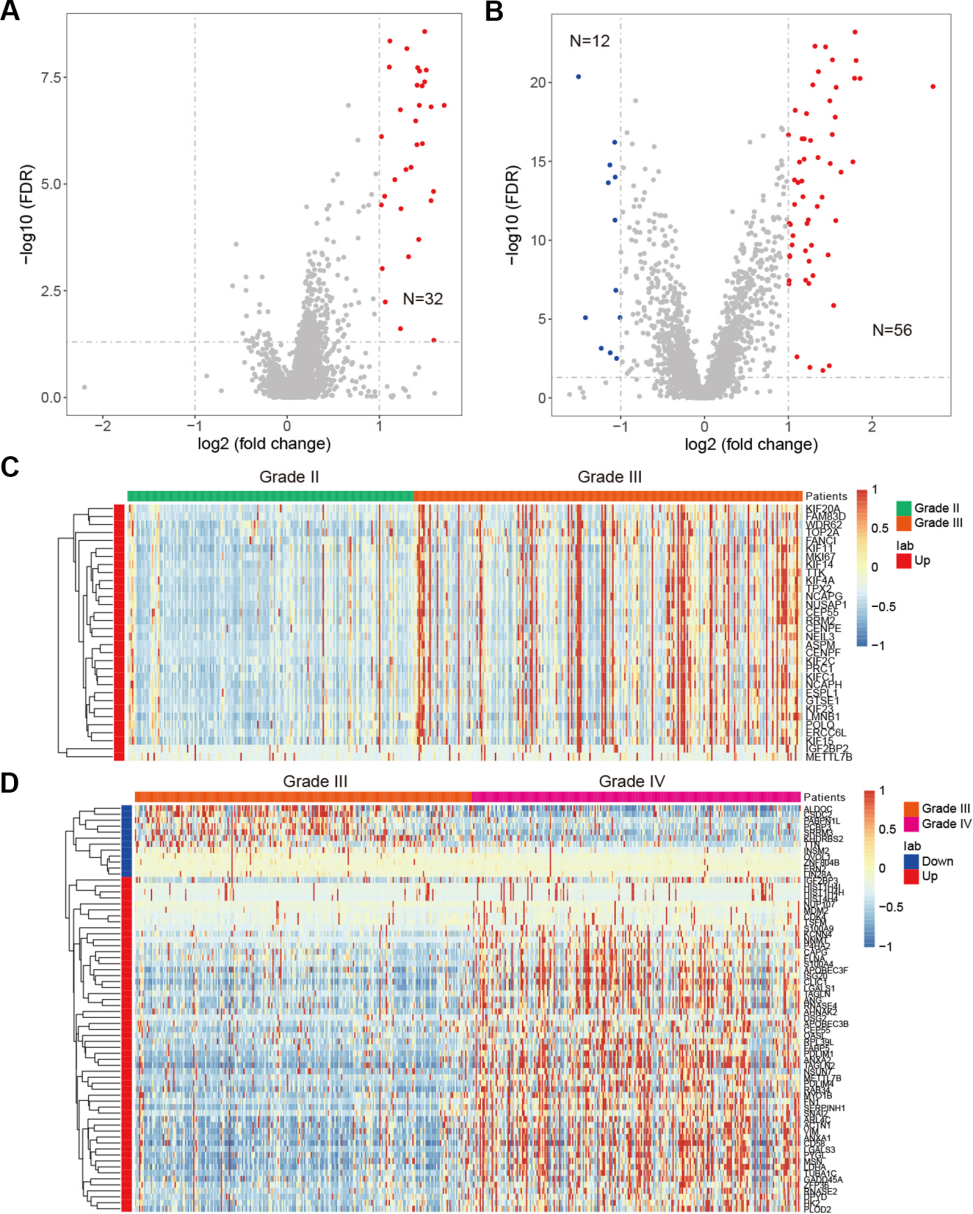
Differentially expressed genes in glioma. **(A)** Volcano plot shows the differentially expressed genes between grade II and III. Red for up-regulated genes. **(B)** Volcano plot shows the differentially expressed genes between grade III and IV. Red for up-regulated genes and blue for down-regulated genes. **(C)** Heat map for genes that perturbed between grade II and III. **(D)** Heat map for genes that perturbed between grade III and IV.

Besides genetic alterations, evidence have suggested that the expression of RBPs were also perturbed in cancer (Sebestyen et al., 2016; Li et al., 2017). We next systematically analyzed the RBP transcriptome in different grades of glioma. We identified 32 up-regulated RBPs in comparison between grade II vs. III (**Figure 5A**). There are more RBPs exhibited expression perturbations when comparison between grade III and IV (**Figure 5B** and **Table S3**). These results suggested that the transcriptome were likely to be perturbed during the progression from low grade to high grade. Among the top up-regulated genes in comparison between grade II and III, we identified four important genes, such as IGF2BP2, TTK, KIF4A and CEP55 (**Figure 5C**). It has been shown that IGF2BP2 was a direct target of miR−188 in glioma, and IGF2BP2 under−expression served tumor−suppressive roles in glioma growth and metastasis (Ding et al., 2017). CEP55 has been found to promote cell proliferation and inhibits cell apoptosis in glioma (Li et al., 2018b).

When we compared the transcriptome of patients in grade III vs. grade IV, we identified 56 up-regulated genes and 12 down-regulated genes (**Figure 5B**). Among the up-regulated genes, we identified NNMT, LGALS3, PDLIM4, TUBA1C and ANXA2 as top five (**Figure 5D**). NNMT silencing had been shown to activate tumor suppressor PP2A and inhibits tumor forming (Palanichamy et al., 2017). This was consistent with our result that it was up-regulated in GBM. LGALS3 was also found to promote GBM and was associated with tumor risk and prognosis (Wang et al., 2019). PDLIM4 had been identified as a gene signature associated with the clinical outcome in high-grade gliomas (de Tayrac et al., 2011). For the down-regulated genes, we have identified CSDC2, ZNF804B, LIN28A and SRRM3 as candidates. However, few of these were investigated in current studies. These results suggested that the tumor suppressors need to be paid attention in future glioma studies. Taken together, function analysis of these RBPs provide insight into the transcriptome perturbations of glioma progression.

### RNA-Binding Protein Regulatory Network During Glioma Progression

**Figure 6:**
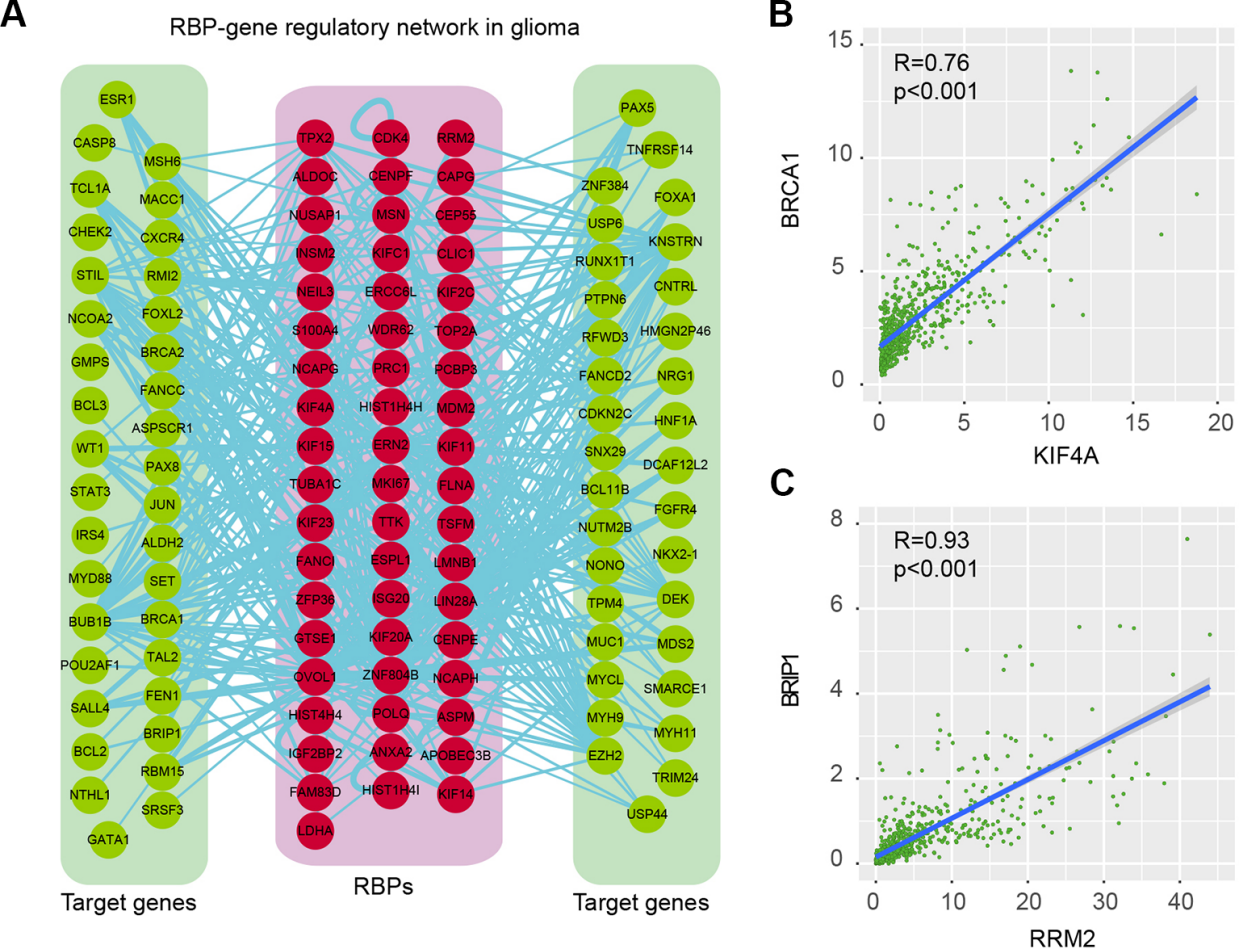
RNA-binding protein (RBP)–gene regulatory network in glioma. **(A)** The RBP–gene regulatory network in glioma. Red for RBPs and green for target genes that were related to cancer. **(B)** Scatter plot shows the correlation between KIF4A and BRCA1 in glioma. **(C)** Scatter plot shows the correlation between RRM2 and BRIP1 in glioma.

Proteins do not function isolatedly but interact with other molecules in complex cellular networks for signal transduction (Xu et al., 2017; Yi et al., 2017). Understanding the RBP regulatory network during glioma progression will get deep insights into their functions. We thus identified the co-expressed genes in glioma. Here, we focused on the cancer-related genes (**Table S4**). At the PCC > 0.70 and *p*-adjusted < 0.05, we identified 368 regulatory interactions among 55 RBPs and 69 genes (**Figure 6A** and **Table S5**). In this regulatory network, several RBPs and genes were correlated with the development and progression of glioma. For example, KIF4A was correlated with cell cycle, G2M checkpoint (Cho et al., 2019). Abrogation of BRCA1 had also been found to play roles in tumor growth in glioma (Rasmussen et al., 2016). We found that the expression of KIF4A and BRCA1 was significantly correlated with each other in glioma (**Figure 6B**, *R* = 0.76 and *p*-value < 0.001), providing a functional link between RBP and gene.

Another example was RRM2 and BRIP1, which was significantly correlated with each other in expression (**Figure 6C**, *R* = 0.93 and *p*-value < 0.001). RRM2 had been found to promote the progression of human GBM and was a potential prognostic biomarker in glioma (Li et al., 2018a; Sun et al., 2019). BRIP1 was found to be an independent signature, which was correlated with worse prognosis in glioma (de Sousa et al., 2017). Our results provided a way to functionally explain the signaling of RRM2 during glioma progression. Moreover, we also identified the functional association between TTK and BUB1B, KIF23 and POLQ. All these RBP–gene correlations provide suitable ways for functional characterization of RBPs in glioma.

### Prognostic Potential of RNA-Binding Protein Regulators

**Figure 7:**
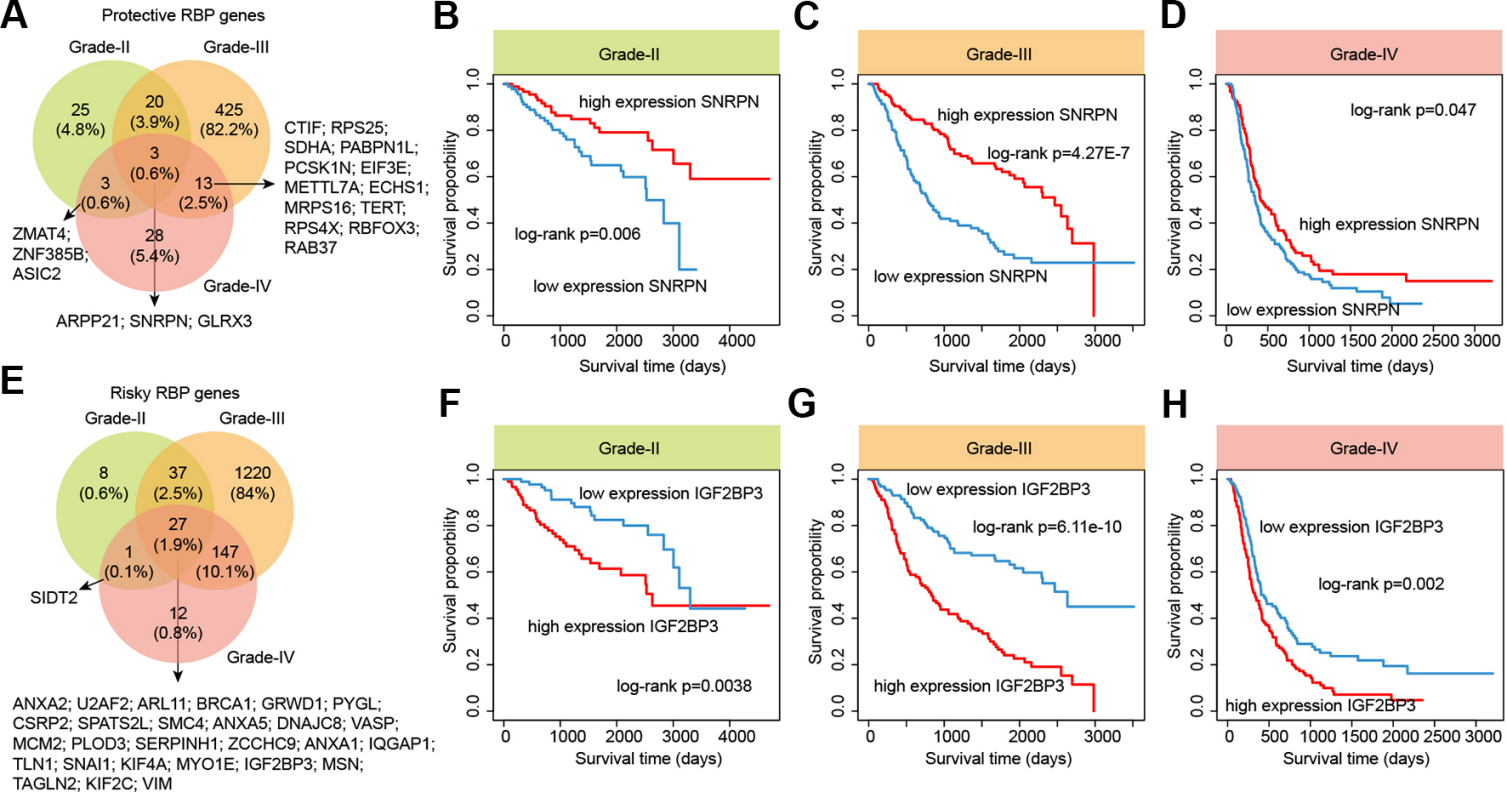
The survival landscape of RNA-binding proteins (RBPs) in glioma. **(A)** Venny plots show the protective RBPs in different grade glioma patients. **(B**–**D)** Kaplan–Meier plots indicating overall survival of glioma patients with low and high expression of SNRPN. **(E)** Venny plots show the risky RBPs in different grade glioma patients. **(F–H)** Kaplan–Meier plots indicating overall survival of glioma patients with low and high expression of IGF2BP3.

RBPs are essential modulators of transcription and numbers of RBPs have being found to be associated with the survival of patients (Frau et al., 2013). We next identified the RBPs that were associated with the survival of patients in different grades. We found that there were more RBPs were associated with survival in grade III, compared with other two grades (**Figure 7** and **Table S6**). For the protective RBPs in glioma, 25, 425 and 28 RBPs were only associated survival in grade II, III and IV. Three RBPs (ARPP21, SNRPN and GLRX3) were associated with patient overall survival in all grades (**Figure 7A**). SNRPN had been found as a autism-related gene by regulating cortical and spine development via nuclear receptor (Li et al., 2016). Hypermethylation of SNRPN increased as the cellular origin of the tumors advanced in oogenesis and was closely correlated in individual teratomas (Miura et al., 1999). We found that the high expression of SNRPN was correlated with better overall survival in all grade glioma patients (**Figures 7B–D**, log-rank *p*-values < 0.05).

In addition, we also identified numbers of risky RBPs in glioma (**Figure 7E**). There were 8, 1,220, 12 RBPs were associated with survival in specific grade. In total, 27 RBPs were identified as risky factors in all grades, including BRCA1, MCM2, IGF2BP3, KIF2C, VIM and PLOD3. High MCM2 expression was found to be strongly associated with poor overall survival in patients with high-grade glioma in our current study, as well as previous studies (Hua et al., 2014). Recently, IGF2BP3 has been identified as a potential oncogene across multiple cancer types (Li et al., 2019b). We found that the high expression of IGF2BP3 was significantly associated with poor prognosis in all three grades (**Figure 7F–H**, log-rank *p*-values < 0.05). These results provided more evidence for the oncogene roles of IGF2BP3 in cancer. Taken together, our analyses provided a prognostic spectrum for RBPs during glioma progression.

## Discussion

In this study, we systematically analyzed the genetic and transcriptome alterations of RBPs during glioma progression. The top mutated RBPs in different grades of glioma patients were identified and several of them had been found to play important roles in glioma or other cancers. Moreover, we compared the transcriptome and identified the differentially expressed RBPs. We found that there were more RBPs exhibited expression perturbations during the transition from grade III to IV. These results suggested that the transcriptome was greatly perturbed in the progression of high-grade glioma. Our regulatory network and prognosis analysis also revealed several important candidates for functional characterization in future experiments.

Although several candidate RBPs were identified in our current study, there are a lot of work need to do for investigating the detail functional ways of these RBPs. RBPs have been found to regulate alterative splicing (AS) and influences the expression of genes (Fei et al., 2017). Alterations of AS are emerging as important signatures in cancer (Liu et al., 2017). The mutation of RBPs generally impair the recognition of regulatory sites, and affecting the splicing of multiple genes. However, it is still difficult to determine the targets for the majority of RBPs. The best method for identifying the targets of RBPs is CLIP-Seq, but there are limited number of data currently. With the development of sequencing technology, such as CLIP-Seq, we will get more details about the function of RBPs. Moreover, RBPs can also interact with noncoding RNAs. Identifying the cell type specific RBP interactome will yield novel insight into the function of RBPs.

Moreover, we identified an important RBP IGF2BP3 during glioma progression. Beside RBP, this genes also an epigenetic regulator, which can affect the fates of mRNA in an m6A-dependent manner (Bi et al., 2019). These results suggest that m6A might also play important roles during glioma progression. Several studies have emerged to reveal the function of m6A in glioma (Dixit et al., 2017; Zhang et al., 2017). But we are still lack of knowledge about the landscape of m6A alterations in glioma, particular in Chinese cohort. Moreover, we are not sure to what extent RBPs can regulate m6A. Is it just one case or general regulation? With the increasing data of RBP regulation as well as other epigenetic data, we will get deep insight into this regulatory layer in cancer.

In summary, our comprehensive analyses dissect the potential function of RBPs during glioma progression. Understanding the functions of candidate RBPs identified in this study will provide insight into the underlying mechanisms of glioma progression.

## Data Availability Statement 

Publicly available datasets were analyzed in this study. This data can be found here: http://www.cgga.org.cn/download.jsp.

## Author Contributions

JW contributed to data analysis and paper writing, JQ contributed to figure construction, XH contributed to the design, data analysis and paper writing.

## Funding

This paper was supported by grant 2015BSB14042 from the Natural Science Foundation of Shandong Province, China.

## Conflict of Interest

The authors declare that the research was conducted in the absence of any commercial or financial relationships that could be construed as a potential conflict of interest.
