# Supplementary material for: Systematically Dissecting the Function of RNA-Binding Proteins During Glioma Progression
Source: Front Genet. 2020 Jan 28;10:1394. doi: 10.3389/fgene.2019.01394 (PMC6997557; doi:10.3389/fgene.2019.01394)
Supplement: Supplementary file 2 [file Presentation_1.pdf]

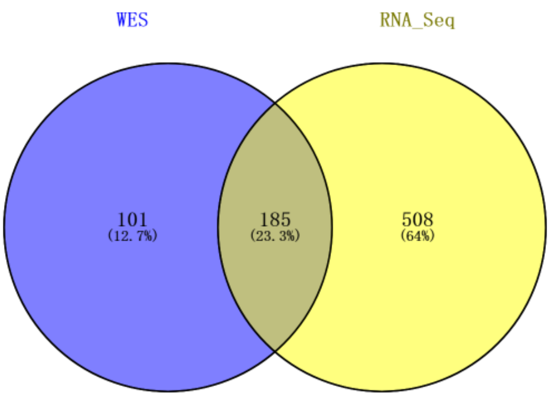

Figure S1. Venny plot showing the overlap of patients with exome sequencing and RNA-Seq data.

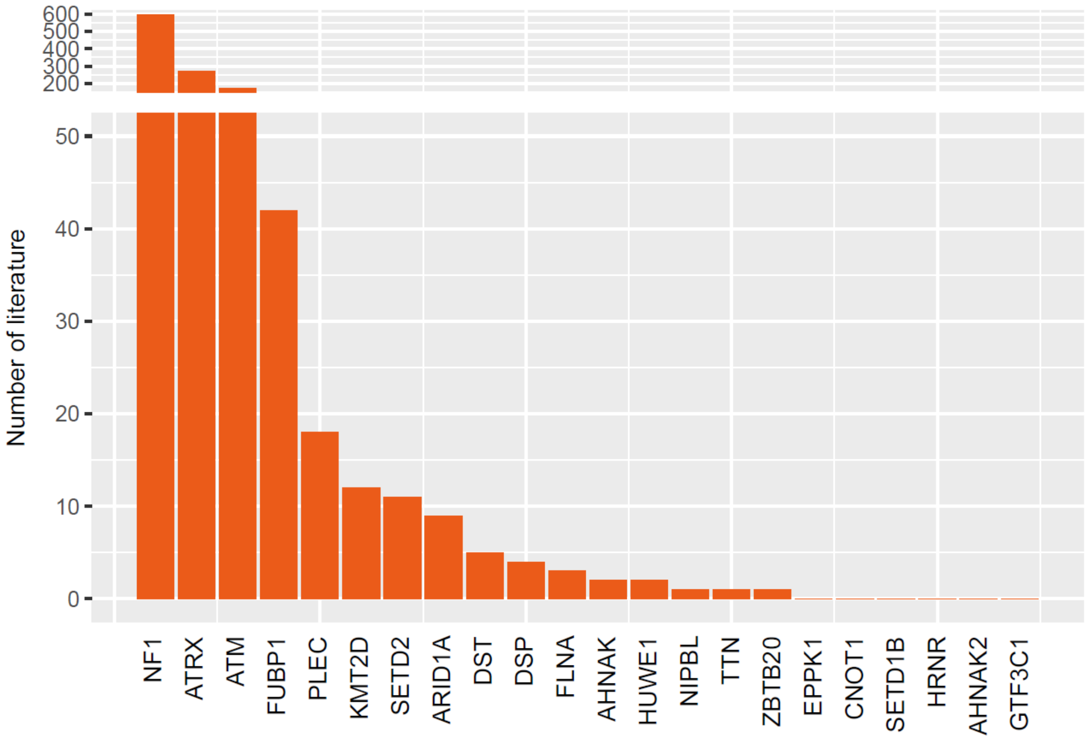

Figure S2. The number of literature genes co-occurred with glioma.

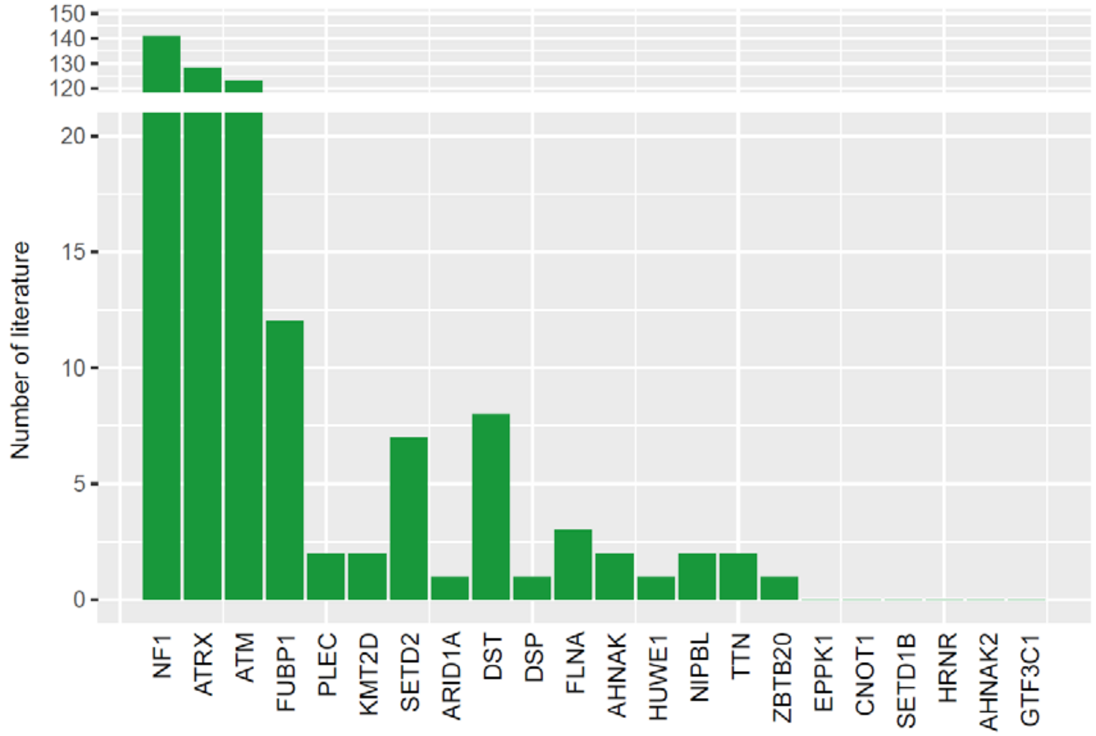

Figure S3. The number of literature genes co-occurred with glioblastoma.
